# An unusual contrast-induced encephalopathy following percutaneous coronary intervention in patients with cerebrovascular abnormalities: A case report

**DOI:** 10.3389/fcvm.2022.957779

**Published:** 2022-11-24

**Authors:** Jiangquan Liao, Yan Wang, Mingjing Shao, Yanling Wang, Jinhang Du, Xianlun Li, Peng Yang, Dongliang Fu, Zhe Dong, Mengru Liu

**Affiliations:** ^1^National Integrated Traditional and Western Medicine Center for Cardiovascular Disease, China-Japan Friendship Hospital, Beijing, China; ^2^Department of Cardiovascular and Respiratory Medicine, Beijing Nuclear Industry Hospital, Beijing, China

**Keywords:** contrast-induced encephalopathy, percutaneous coronary intervention, blood-brain barrier, hypothyroidism, cerebrovascular abnormalities

## Abstract

**Introduction:**

Contrast-induced encephalopathy (CIE) is a complication associated with the administration of iodinated contrast, which usually happens minutes to hours after contact with contrast, and fully recovers within 72 h. The clinical manifestations of CIE are diverse, and the pathological mechanism is not explicit.

**Methods:**

We report the case of a 66-year-old female who suffered from a delayed CIE following the administration of iodinated contrast agent. Symptoms were severe. Imaging examination, biochemical and etiological detection were performed timely. The course of neurological symptoms was atypical. Her complex complications of hypothyroidism and cerebrovascular abnormalities contributed to more challenges, which were also clues to the diagnosis. With prompt and active treatment, the patient recovered fully over 10 days.

**Discussion:**

The diagnosis standard of CIE highly depends on the association with the contact of contrast and the exclusion of other nervous system diseases. Complicated clinical circumstances and individual specificity can lead to different clinical manifestations of CIE, making it even more difficult to diagnose and treat. Prompt and dynamic imaging examination would provide great value in the diagnosis and evaluation of CIE. Timely diagnosis and intervention may be the key to its satisfying prognosis.

## Introduction

Contrast-induced encephalopathy (CIE) is a rare neurological complication associated with the intra-arterial administration of iodinated contrast. The incidence of CIE associated was estimated between 0.05 and 0.11% for diagnostic intravascular coronary angiography (ICA) and between 0.3 and 0.4% for percutaneous coronary intervention (PCI) (1). Its clinical manifestations are diverse, and the diagnosis of CIE is usually exclusionary (2). CIE can lead to various neurological symptoms within minutes to a few hours after the injection of contrast. Most of the symptoms would disappear with the removal of the contrast agent by kidney within 72 h. With prompt and active treatment, sequela usually does not occur. Herein, we present a patient with cerebrovascular abnormalities who suffered from a delayed CIE following PCI and fully recovered over 10 days.

## Case report

A 66-year-old female was hospitalized due to 1 month of chest tightness and unexplained medium pericardial effusion diagnosed 1 week before admission by echocardiogram. The previous history includes kidney trauma and hematuria caused by an impact 3 years ago, with no sequela. The blood test indicated cardiac troponin T (cTnT) 0.117 ng/mL (normal range < 0.014 ng/mL). Electrocardiogram showed T wave inversion in V4-6. ICA was performed through the right radial artery, revealing moderate to severe stenosis in the left anterior descending branch (LAD), and one drug-eluting stent (DES) was implanted according to standard procedure. There is no complication during the PCI procedure. Approximately 100 ml of iopromide (62.34 g iopromide per 100 ml) (Bayer Pharma AG), a non-ionic hypotonic contrast agent, was administered during the procedure. Local anesthetic (1% lidocaine) was administered prior to ICA, and 6,000 iu intra-arterial heparin was administered during PCI.

Blood tests reported later that day showed that the thyroid function was very low, with free thyroxine 4 0.12 ng/dL (normal range 0.93 to 1.7 ng/dL), free thyroxine 3 0.27 pg/mL (normal range 2.0 to 4.4 pg/mL), thyrotropin 63.87 uIU/mL (normal range 0.27 to 4.2 uIU/mL), and thyroid antibody high off the chart. We considered hypothyroidism resulting from Hashimoto’s thyroiditis. Other abnormalities in blood tests included serum creatinine 123.5 umol/L (normal range 63.5 to 106 umol/L), Na + 131 mmol/L (normal range 135 to 145 mmol/L), total cholesterol (TC) 7.45 mmol/L (normal range < 1.7 mmol/L), and low-density lipoprotein cholesterol (LDL-C) 4.1 mmol/L (normal range < 3.4 mmol/L). Dual antiplatelet (aspirin 100 mg/d and clopidogrel 75 mg/d), enhanced lipid-lowering (atorvastatin 20 mg/d and ezetimibe 10 mg/d) were administered.

**FIGURE 1:**
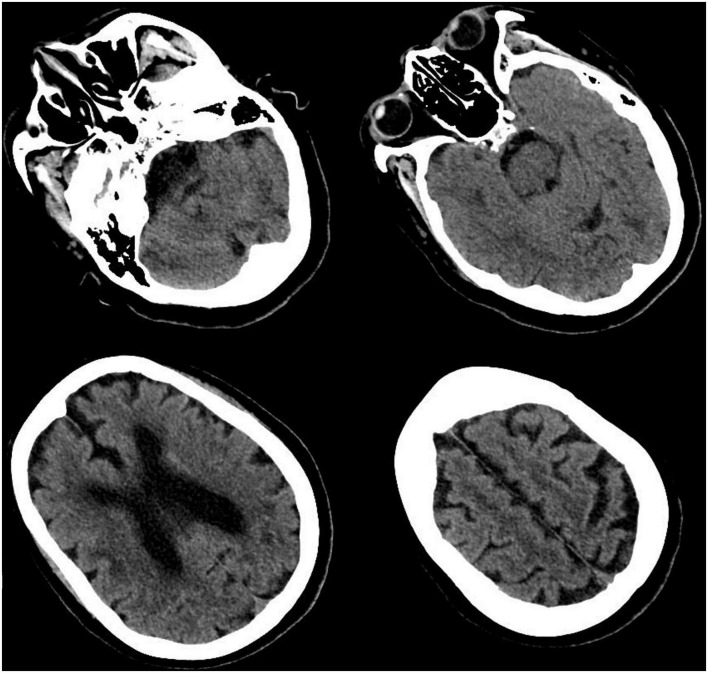
Cranial computed tomography (CT) scan. CT scan showed multiple cerebral infarction, white matter degeneration, brain atrophy, with no sign of cerebral hemorrhage or space-occupying lesion.

**FIGURE 2:**
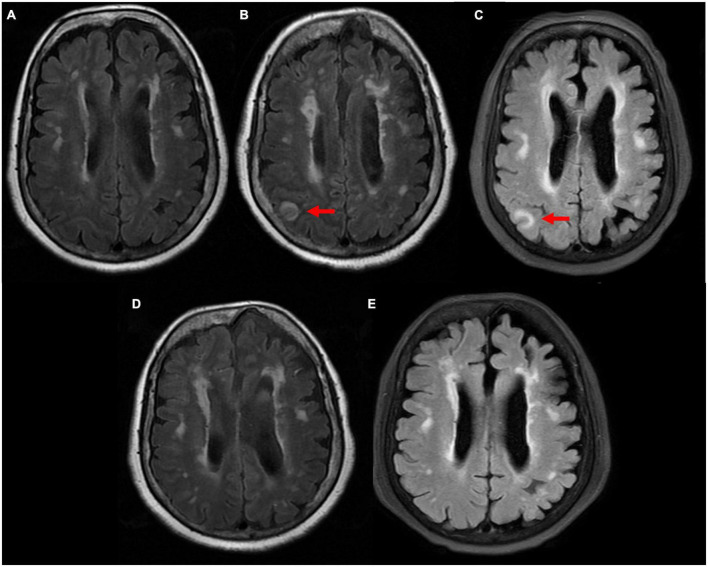
Comparison of magnetic resonance imaging (MRI) T2 flair sequence. **(A)** 4 years ago patient suffered from moderate headache and performed MRI scan in our clinic department. It showed multiple ischemic infarcts, white matter degeneration, brain atrophy, with no sign of abnormal signal. **(B)** 24 h after neurological symptoms, right parietal and occipital cortex swelling with abnormal signals was observed. **(C)** 72 h after neurological symptoms, right parietal and occipital cortex swelling with abnormal signals remained. **(D)** 12 days after neurological symptoms, abnormal signals disappeared. **(E)** 50 days after neurological symptoms, no sign of new abnormal signal.

**FIGURE 3:**
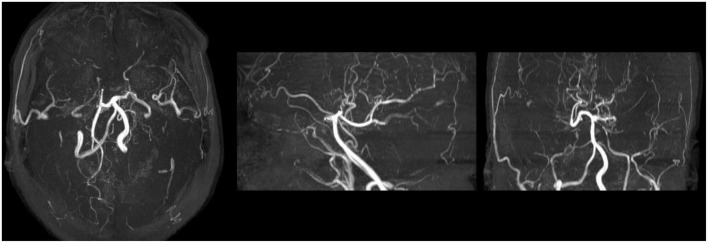
Maximum Intensity Projection sequence of MRA scan after neurological symptoms. MRA scan showed distal internal carotid artery occlusion, bilateral anterior and middle cerebral arteries occlusion, abundant and disordered collateral circulation at the circle of basal artery and bilateral basal ganglia, considered moyamoya disease.

Forty-eight hours after PCI, she began to suffer from severe headaches, speech disorder, trance, uncooperative in physical examination, and slow pupil light reflex. Cranial CT scan was performed immediately and showed no evidence of intracranial hemorrhage but multiple old infarcts, white matter degeneration, and brain atrophy (Figure 1). About 62 h after PCI, her temperature began to rise and deteriorated into loss of consciousness, eyes stared to the right, dull pupil light reflex, no response to pain stimulation of limbs, spasticity, and suspicious positive pathological signs of the left lower limb. Urgent brain MRI showed multiple ischemic and infarct foci, right parietal and occipital cortex swelling with abnormal signals, expansion of supratentorial ventricular system, and degeneration of brain white matter (comparison of MRI is shown in Figure 2). Blood tests revealed no significant changes. Lumbar puncture and cerebrospinal fluid examination, antinuclear antibody spectrum test, electroencephalogram were conducted, but no results with clear diagnostic significance were found. Cranial MRA showed distal internal carotid artery occlusion, bilateral anterior and middle cerebral arteries occlusion, abundant and disordered collateral circulation at the circle of the basal artery and bilateral basal ganglia, all of which were classified as moyamoya disease (Figure 3).

In other hospitals, a family member provided a medical history of an uncertain diagnosis of congenital cerebrovascular dysplasia with an uncertain imaging examination. Multidisciplinary consultation was conducted, and myxedema coma caused by hypothyroidism, brain edema caused by hyponatremia, intracranial infection, brain tumor, and other nervous system diseases were excluded. The diagnosis of CIE was performed. The treatment of CIE was engaged during these examinations. Dexamethasone, mannitol, parenteral nutrition solution, thyroxine, and glucose sodium chloride supplement were performed.

Seventy-five hours after the onset of these symptoms (5 days after PCI), the patient regained consciousness and was capable of simple conversation. Ten days after her consciousness, she had fully recovered and discharged from the hospital. During her last 10 days in the hospital, her physical activity gradually recovered, but she experienced dysphoria and disorientation several times. Two weeks after discharge, she returned to the outpatient clinic with no sign of discomfort. Physical and chemical examinations were within normal range. Echocardiogram showed no sign of pericardial effusion. MRI showed that the right parietal and occipital cortex swelling with abnormal signals disappeared (comparison of MRI is shown in Figure 2). Yet she has lost memory from the onset of neurological symptoms to fully recover.

## Discussion

Contrast-induced encephalopathy was first reported in Fischer-Williams et al. (3), which is a rare complication of the administration of contrast agent. The clinical manifestations of CIE are different signs of neurological dysfunction which usually are transient. CIE is sometimes indefinitely diagnosed, requires admission of an iodinated contrast agent, a short interval between exposure to contrast and onset of symptoms, full recovery from neurological symptoms within days, and is excluded from other pathological processes. CIE happens 0.5 to 18 h after the administration of the iodinated contrast agent, according to present reports (4). With or without treatment, symptoms usually resolve spontaneously within 48 to 72 h, as the contrast agent is eliminated by the kidneys (5). Typical radiological findings of CIE include cerebral edema and cortical enhancement.

The pathological mechanism of CIE is not explicit. Current understanding of CIE is the dysfunction of the blood–brain barrier (BBB) and neurotoxicity caused by iodine-based contrast. Under normal conditions, the BBB can block the iodinated compounds out of the nervous system. When the integrity of the BBB is disrupted and the contrast agent permeates the central nervous system, it causes injury through direct neuronal toxicity (1). The high iodine concentration in contrast, increased neuronal excitability due to receptor activation, and lipid solubility of the contrast medium have all contributed to the contrast’s neurotoxicity (6–8).

The main risk factors of CIE are not certain due to the lack of the statistic data. Renal dysfunction (decreased glomerular filtration rate) as one of the major risk factors is well-established (9, 10). Other than that, previous stroke and heart failure are major risk factors for CIE (11).

In this case, there were many unusual features we can find during the procedure. First, the neurological symptoms happened 48 h after the administration of the contrast agent, which was longer than ever reported cases of CIE. Second, the neurological symptoms were fully resolved but had lasted for over 10 days. Other than these, she was accompanied by renal dysfunction, hypothyroidism, hyponatremia, pericardial effusion, and moyamoya disease. Among these diseases, moyamoya disease (MMD) is a chronic occlusive cerebrovascular disease characterized by partial progressive stenosis at the end of the internal carotid artery and abnormal vascular network at the bottom of the brain (12). Narducci et al. (13) first demonstrated that the BBB was impaired in patients with MMD with a retrospective study.

With all these leading evidence, we supposed the following pathophysiological process. Following contrast agent administration, the BBB, which had been compromised by MMD, was unable to keep the contrast out of the nervous system. Contact with iodine-based contrast led to neurotoxicity. Hyponatremia, attributed to hypothyroidism, deteriorated the impairment of BBB, and cerebral edema. Low metabolic level and renal dysfunction which are attributed to hypothyroidism had led to a delayed elimination of contrast. These may explain the delayed and prolonged procedure of CIE, and the changes in imaging abnormalities abided by the symptoms. Lateral evidence for the diagnosis of CIE are that we treated her with a standard treatment of CIE, including dexamethasone, mannitol, and nutrition support. Biochemical and etiological detection of cerebrospinal fluid did not return diagnostic results. Brain tumor was also excluded after the re-examination of MRI. Hence we have strong confidence that this is the first reported delayed CIE following PCI in patients with cerebrovascular abnormalities.

In conclusion, CIE is a rare complication of the administration of iodinated contrast agent, with varied clinical manifestations. The diagnosis standard of CIE is not definite, which highly depends on the association with the contact of contrast and the exclusion of other nervous system diseases. Prompt and dynamic imaging examination would provide great value in the diagnosis and evaluation of CIE. The pathological mechanism of CIE is not explicit, which leads to only supportive treatment, not etiological treatment in clinical practice. Complicated clinical circumstances and individual specificity make it even more difficult to handle. Timely diagnosis and intervention may be the key to its satisfying prognosis.

## Data availability statement

The original contributions presented in this study are included in the article/supplementary material, further inquiries can be directed to the corresponding author.

## Ethics statement

Ethical review and approval was not required for the study on human participants in accordance with the local legislation and institutional requirements. The patients/participants provided their written informed consent to participate in this study. Written informed consent was obtained from the individual(s) for the publication of any potentially identifiable images or data included in this article.

## Author contributions

JL, YW, and MS supervised and revised the manuscript. YLW, JD, and XL drafted the manuscript. PY, DF, ZD, and ML processed the clinical information and imaging. All authors contributed to the article and approved the submitted version.
